# Reaching out or missing out: approaches to outreach with family carers in social care organisations

**DOI:** 10.1111/hsc.12119

**Published:** 2014-10-21

**Authors:** Jo Moriarty, Jill Manthorpe, Michelle Cornes

**Affiliations:** Social Care Workforce Research Unit, King's College LondonLondon, UK

**Keywords:** carers, equalities, exclusion, information, interviews, outreach, social care, stigma, survey

## Abstract

Outreach is advocated as a way of improving the uptake of services among underserved populations and of filling the gaps between mainstream services and the populations they are intended to support. Despite the policy emphasis on providing better help for family carers, research consistently shows that many of those providing unpaid care to a family member or friend report difficulties in finding out about the assistance to which they are entitled. This article presents results from a concurrent mixed-methods study, which aimed to describe different ways of working with family carers in adult social care departments and to collect the views of a range of stakeholders about the advantages and disadvantages of the approaches that were identified. A total of 86 semi-structured face-to-face interviews were undertaken with a purposive sample of funders, carers' workers, representatives of voluntary organisations and family carers based in four contrasting localities. An email survey was sent to all local councils in England with social care responsibilities and resulted in a 53% response rate. Data collection took place in 2012, with a small number of interviews being completed in 2011. Our approach to data analysis combined methodological, data and theoretical triangulation. The findings presented here mainly draw on the interview data to highlight the different models of outreach that we identified. The article highlights important differences between outreach and the provision of information. It concludes that organisations providing support for carers need to consider the advantages and disadvantages of different models of outreach as they develop carers' support and the extent to which different models might be more effective than others in reaching particular types of carer.

**What is known about this topic**

- Many carers report difficulties in finding out about services, meaning that they are sometimes caring without any other support.
- Barriers to accessing support include stigma, guilt and not recognising oneself as a carer.
- Outreach services have been widely developed as a way of reaching out to under-represented or at-risk groups, but there is a lack of clarity and precision about what constitutes outreach.

**What this paper adds**

- Information services on their own are insufficient in ensuring that all carers can find out what social care support is available and how to access it.
- There are different models of outreach work with carers, but further research is required to identify which models work most effectively, in what circumstances and for whom.
- Information and outreach strategies need to become more tailored to reduce the number of ‘hidden carers’.

## Introduction

Rises in the number of older adults and people with disabilities needing support and better recognition of the negative consequences that prolonged intensive caring can have for many carers (Moriarty 2012) have created increased pressures on governments across the more developed and developing world to find better ways of supporting those providing unpaid care to family members and friends. Researchers have drawn attention to the comparatively well-established legislative and policy framework in England that gives carers specific rights (Glendinning 2003, Parker *et al.* 2010, Courtin *et al.* 2014). Insofar as local councils with social services responsibilities are concerned, these include the statutory obligation to inform carers providing ‘regular and substantial’ care (for which there is no set definition) of their rights to an assessment and to ensure that the assessment considers their opportunities to take part in education, training, employment and leisure activities. However, the number of carer assessments continues to be low and they often fail to explore adequately carers' abilities and willingness to continue caring (Mitchell *et al.* 2013).

Councils do not yet have a duty to provide services for carers (although they will do once the Care Act 2014 is implemented). This Act also places new duties on every council with social care responsibilities to ‘establish and maintain a service providing people with information and advice relating to the care and support available in the locality’ and removes the requirement that only carers giving ‘regular and substantial’ care qualify for an assessment.

In this context, it seems timely to consider how councils currently help carers access information about their entitlements. Apart from the Mitchell *et al*. (2013) study of carers and personalisation and a survey of carers who had received a carer's assessment or cared for someone whose support needs had been assessed or reviewed by their local council in the previous year (Fox *et al.* 2010), much of the existing published evidence consists of data collected over a decade ago (e.g. Arksey 2002, Seddon *et al.* 2007). Since then, there have been major changes in the way that social care is provided, exemplified in the policy of personalisation, which aims to optimise the choice and control that people can exercise over their social care support. Strikingly, as Glendinning *et al*. (2013) point out, the twin policies of personalisation and support for carers seem largely to have developed independently of each other.

Perhaps even more importantly, most research on social care support for carers was undertaken before the vast majority of local councils implemented real-term cuts in their social care expenditure. When these cuts are combined with steep increases in the numbers of older people and adults with disabilities needing social care (NHS Confederation 2012, Local Government Association 2013), it becomes apparent that social care support for carers is taking place in a very different environment from the one in which the previous Labour government's Carers' Strategy (HM Government 2008) promised carers ‘a caring system on your side, a life of your own’.

### How do carers access support?

Despite their legal rights to support, carers consistently report difficulties in finding out what help is available and receive very little assistance (Carers UK 2013). Carers identified from local council records are older and spend more time caring per week compared with the wider population of carers (NHS Information Centre for Health and Social Care 2010). Increasing attention is also being given to ‘hidden’ or under-represented carers (Cavaye 2006) who seem even less likely to access support. Examples include carers from black and minority ethnic groups (Katbamna *et al.* 2004, Milne & Chryssanthopoulou 2005, Merrell *et al.* 2006), lesbian, gay, bisexual and transgender carers (Willis *et al.* 2011), young carers (Children's Society 2013) and working age carers in paid employment (King & Pickard 2013).

### Outreach

One response to situations in which particular groups have been identified as less likely to use services – thus becoming ‘underserved’ – has been through the use of outreach. As Dewson *et al*. (2006, p. 1) observe, ‘outreach is a term that is often used but rarely explained’. Citing McGivney (2000), they argue that their own literature review confirmed her earlier findings that its meaning ‘appears to be taken for granted’ or alternatively that terms such as ‘engagement’ or ‘community development’ are used instead to describe outreach activities (Dewson *et al.* 2006, p. 11). A further complexity when considering outreach from an international perspective is that similar approaches may exist across countries, but their precise forms and the terminology used to describe them differ (Kloppenburg & Hendriks 2012).

Andersson (2013) agrees that the term lacks clarity and precision, but proposes a broad definition:

The fundamental idea of outreach work is to start a process of social interaction between people in need, on the one hand, and some kind of support-oriented organizational body on the other.

(p. 5)

He suggests that one factor hindering the development of a universally agreed definition stems from the context-specific nature of outreach. Most published research concentrates on outreach with stigmatised groups, such as sex workers (Coy 2006) or homeless people (Jost *et al.* 2010), sometimes with the intention of making changes to, or controlling, recipients' behaviour. There is another literature based on health promotion outreach with groups that are either under-represented in services compared with epidemiological estimates of their need and/or that have been identified as being at greater risk of a particular condition (Ahmad *et al.* 2013, Whitney *et al.* 2013). Looking across both types of study, the overall quality is variable and there has been a lack of progress in identifying the effectiveness of different models of outreach with specific outcomes (Dewson *et al.* 2006, Andersson 2013, Whitney *et al.* 2013).

In the context of caring, carers' centres theoretically provide an avenue from which to explore outreach. Often located on high streets or in shopping centres, they encourage carers to ‘drop in’ without an appointment and are run by independent charities. However, published evaluations of carers' centres tend to focus on different outcomes, such as their role in maintaining carers' mental health (e.g. Clifford *et al.* 2011).

It is rare, too, to find published examples of other types of outreach work with carers. Hughes *et al*. (2011) focused on carers' experiences of an assertive outreach team. While team members and carers both shared the view that carers benefited from the service, equally they acknowledged that the team's prime focus continued to be on supporting people with a mental health problem and not on their carers.

Some services are specifically designated as ‘Carers Outreach’ (e.g. Alzheimer's Society 2014), but a lack of published comparative research means that it is not clear whether they operate differently from other types of information and advice service.

This present article has two aims. The first is to describe some of the information strategies used by local councils to identify carers living within their locality. The second compares some of the advantages and disadvantages of different models of outreach that we identified from the perspective of service providers, workers, representatives of voluntary organisations and family carers. We use these findings to argue that the diversity that exists among those caring for a family member or friend means that local councils will need to develop multiple strategies to maximise the success of the information services they provide for carers.

## Methods

### Design

The study adopted a concurrent mixed-methods design based on data from face-to-face semi-structured interviews undertaken in four different parts of England and email/postal responses to a survey sent to all adult social care directors. Mixed-methods designs are increasingly popular, mainly because they offer both breadth and depth of information about a particular topic (Johnson *et al.* 2007). This is thought to be especially useful when the topic has been under-researched (Cresswell & Plano Clark 2011). Although the survey responses did produce some quantitative data, this was primarily a qualitative study, as befitted one in which existing research is quite limited and where there was an emphasis on capturing the subjective or ‘lived experiences’ of different types of participants (Marshall & Rossman 2011).

### Sample selection

Four different adult social care departments in England were requested to take part in the study. They were selected on the basis of maximum variation sampling in which there is a deliberate intention to include phenomena that differ widely from one another (e.g. geographical location, and population size and composition). This approach helps to identify whether there are central themes that cut across participants, organisations or localities (Patton 2002).

### Interview data

We used a combination of purposive and emerging sampling to select a group of participants likely to be ‘information-rich’ (Patton 2002, p. 46) about support for carers in their locality. Using carers’ directories developed by local health and care services and other resources, we identified commissioners responsible for planning support for carers (*n* = 8), and representatives of voluntary organisations supporting carers or people likely to have carers (*n* = 16). We asked these informants to put us in touch with family carers (*n* = 24) and with workers within their organisation whose job description included a specific remit to support family carers (*n* = 38). It was a specific requirement of ethical approval that carers' workers and carers were not to be approached directly by the research team to minimise any pressure that they might feel to participate. Once data collection was underway, multiple operational constructs were used to ensure that we captured perspectives on differing types of caring (e.g. caring for a person with a mental health problem or caring for a partner). The interviews were carried out face to face using a semi-structured schedule that combined exploratory and hypothesis-testing approaches (Kvale & Brinkmann 2009) and was informed by existing research and emerging policy debates. Interviews lasted, on average, 50 minutes.

### Survey data

The survey was attached to an email request sent to all directors of Adult Services departments in England, asking them to pass on the survey for completion by the Carers Lead or other person responsible for their policy on carers. The survey consisted of a short mixture of open and closed questions designed to elicit information about local services for carers and priorities for improvements. Respondents were given a choice of replying by email, post or taking part in a telephone interview, with the overwhelming majority (89%) responding by email. Three reminders were sent between February and May 2012. Overall, a total of 80 replies were received, representing a 53% response rate.

### Data analysis

Interview data and responses to open-ended survey questions were analysed using QSR NVivo 10 (QSR International 2012) using a process of applied thematic analysis (Guest *et al.* 2012). Thematic analysis focuses on identifying and describing both implicit and explicit ideas within the data to generate themes. These themes were partly driven by the literature (e.g. outreach location) and partly driven by the data (e.g. outreach work with other professionals). Numeric data from the study were entered into IBM SPSS Statistics (version 21) (IBM SPSS Statistics, 2012). By comparing the different types of data (survey and interview data), data across different informants (survey respondents, commissioners, carers, carers' workers and voluntary organisations) against existing research on outreach, we aimed to achieve methodological, data and theoretical triangulation (Seale 1999).

### Ethical approval and consent procedures

Ethical approval was received from the Social Care Research Ethics Committee. The Association of Directors of Adult Social Services gave their support to the study. Research governance permission was sought in the four areas taking part in the interviews. All those interviewed gave their informed consent. For survey respondents, the survey information sheet explained that return of a completed survey implied consent to participate. The names used are pseudonyms.

## Findings

### Funding uncertainties

It is important to preface our findings by drawing attention to the impact of fiscal austerity and increased demand for social care support. Almost all the interview participants and survey respondents referred to organisational or funding changes that were thought to have implications for the service they provided, although – as these extracts show – those who were responsible for commissioning the whole of adult social care and those who were working directly with carers faced different pressures:

I certainly wouldn't say this on the record if I didn't know this was going to be anonymous, but I think it's going to be very difficult for councils to do anything other than what they statutorily have to do.

(Delia, Commissioner 6)

And if they do start all these cutbacks, I am actually really concerned what's going to happen to the carer? … And I do get frustrated [that] … services are cut where they really are desperately needed. I get extremely frustrated.

(Olwen, Worker 16)

### Identifying and informing carers

Over 80% of survey respondents reported that their organisation, or another local one, maintained a Carers Register. These registers were used to send information to carers, to consult with and update them on developments locally, and to help councils plan and improve services.

Both survey respondents and interview participants described how leaflets for carers provided in local libraries, contact centres and other venues or information on council websites were accompanied by national and local awareness-raising efforts. For example, Carers Week (an annual event held throughout the UK aimed at raising awareness of carers and helping the public identify themselves as carers) was an important event in the calendar for more high-profile efforts to engage with carers in the locality, such as setting up temporary information stalls or carers' buses in town centres.

Attempts were also made to identify carers more proactively. One commissioner reported they were working with a major supermarket to recognise carers, for example, by identifying customers doing two sets of shopping or assisting another individual to do their shopping:

We are keeping an eye on the pilot work that's going on down in [city] with [supermarket] identifying carers … [Supermarket] … have trained up their till operators to ask the question if there are, say, two people going through checkout and they say, you know, ‘Are you a carer for this individual?’

(Desmond, Commissioner 7)

However, family carers and carers' workers pointed out that information, in itself, was not always sufficient. Both types of participants considered that carers often needed more specialist and timely information than was provided in leaflets or on websites. These latter vary considerably in their content, as we have reported elsewhere (Manthorpe *et al.* 2013). This viewpoint was typified in an interview with a mother who cared for her daughter with mental health problems:

INT: If you wanted more information, would you look on the local authority website?

RES: I wouldn't now, because [daughter's] all right. It's when she's not all right that I need somebody.

INT: Where would you look if you wanted information?

RES: I don't know. I just wouldn't think of it. I think I'm in too much of a state [then] to even use the computer to be honest. (Wilma, Carer 22)

### Carers' centres and other access points

Eighty-five per cent of survey respondents reported that there was a carers' centre in their locality. Few councils ran their own, but instead contracted with voluntary organisations, usually Network Partners of the Carers' Trust, although some were turning to other organisations or consortia to run Carers Hubs.

In one urban area, a manager highlighted attempts to improve the centre's physical and online visibility:

We made the centre a [brighter] much more approachable place to look at … We've [also] done a huge amount of work around our own website. We brought a forum of carers together and, as a result of that, have got a really easy user-friendly website that [works] for carers, as well as professionals.

(Natalie, Worker 14)

However, the option of a designated carers' centre was not always feasible in more rural localities where peripatetic approaches to outreach were more common. The Chief Executive of a rural voluntary organisation highlighted the challenges where transport links were poor and where carers were geographically dispersed:

We have drop-ins in church halls … and they are not always successful, to be honest. You can have somebody sitting there for a day and nobody comes … If we can have more of a road show, if you like, a rolling programme of events that happened around the villages and smaller towns, [then that] then makes the service more accessible.

(Kathleen, Vol 13)

### Integrated outreach in primary care

The advantages and disadvantages of integration between health and social care services in England have been debated regularly over the years. There is now greater policy emphasis on integration as a driver towards service improvements. Most of the interviews were carried out in early 2012 when participants were unclear if the NHS reforms taking place in April 2013 would impact on social care support for carers, but one commissioner described a pilot in which Adult Social Care and primary care had worked together to improve the way they identified family carers:

We have had for a while now … a carers' support worker attached to the GP practices in [Town] and part of their role was to help GPs identify carers and a recent survey of the carers' registers in the GP practices has sort of indicated that those practices that engaged most with that support worker have a higher number of carers on their register than those that were less engaged … We don't know whether that's because they are more carer aware … or whether the carer support worker has raised that awareness. We are trying to establish which it is. Our assumption is that the carer support worker has raised the awareness, but we have got to prove that.

(Hilton, Commissioner 8)

Another participant, part of whose role was to act as the ‘carer lead’ within a GP practice, suggested that such an approach might work better with some older carers:

…and they would perhaps see a social worker coming in as quite stigmatising because … social worker[s] … deal with social problems … We see it with other elderly couples who won't accept help.

(Blythe, Worker 34)

### Self-help outreach

A small number of studies have explored peer support and self-help interventions as a means of improving carers' social support and well-being (Munn-Giddings & McVicar 2007, Charlesworth *et al.* 2011), but do not appear to have considered their role in outreach. One carer participant had set up a group over 10 years ago. He advertised it using leaflets and posters in his local GP's surgery and personal contacts and received a small annual grant from the local council to help with its running costs. He wondered whether his own determination to identify carers and its informal status had been an advantage:

Carers are hard to find … It's just a question of talking and talking and more talking until I eventually found one and I found another one and then it spread from there, really … I don't know whether you get more [carers] from the informal friendliness than you [would] do from the bureaucratic side coming in … A lot of people do hesitate as soon as you say social services and it's got a bit of a stigma attached to it … Fellow carers have been there, seen it and done it. You have opened up another avenue and you've got a friend and you've got a possible contact and a lifeline.

(Maurice, Carer 18)

### ‘Hidden carers’ and the role of specialist outreach

While the overwhelming majority of survey respondents maintained Carers Registers, as mentioned earlier, they also recognised that the few hundreds or thousands of carers on these registers represented just a small proportion of all those caring in their locality.

To a large extent, this disparity could be explained by the phenomenon repeatedly reported in the care-giving literature (O'Connor 2007) – namely that carers only come forward to ask for help if they recognise themselves as carers:

I sometimes think people don't recognise that they are carers themselves, even though they maybe kind of know they are, but they are so busy just doing that role that they don't always see themselves as that person.

(Kevin, Worker 2)

This extract resonates with earlier findings about the context-specific way in which carers absorb and process information, exemplified in Wilma's comment that when things were going well, she did not identify herself as a carer and, when things were more difficult, she felt too overwhelmed to look for information on the internet.

However, there was also a consensus that certain types of carers were even less likely to come forward and ask for support. When asked to select three types of carers that they had most difficulty in identifying, over half of the survey respondents identified carers from black and minority ethnic groups as the main group, followed by working age carers and lesbian, gay, bisexual and transgendered carers.

Lack of suitable information and stigma are regularly identified as reasons why carers from black and minority ethnic groups (Katbamna *et al.* 2004, Moriarty *et al.* 2011) or young carers (Gray *et al.* 2008) are under-represented among those using services. In their study into uptake of mammography services by South Asian women in Canada, Ahmad *et al*. (2013) distinguish between information delivered by indirect and direct mode. They use the former to refer to written or broadcast material aimed at the target community as a whole, while the latter describes:

Structured awareness-raising activities in which messages are communicated face-to-face by socioculturally competent professionals or trained peers.

(p. 91)

A worker who spoke several community languages highlighted that while access to socioculturally appropriate information and translated materials could help, their usefulness was limited in the absence of ‘direct mode’ information:

…trying to get [this carer] to understand the terminologies that are being used … is really difficult on the phone. Hence [I am] going to … take … leaflets that have information about the diagnosis that [her husband] has … I think I need to go and do a home visit and sit down and do a face-to-face and get her to understand a little bit.

(Ifrah, Worker 20)

Furthermore, ‘stigma’ was described as being much more pervasive than in relation to carers from black and minority ethnic groups or young carers. In addition to the stigma around using social care services mentioned earlier, carers of people with substance misuse and, to a lesser extent, carers of people with an eating disorder could also be deterred from seeking support from mainstream services:

…people in these situations can feel that they're very isolated, can feel a lot of stigma around this … and so it very much helps them to know there are others in a similar position … Part of it is just the general society stigma [towards people who misuse substances], but another part of it is that parents often feel responsible for their children and parents of women and men who use substance misuse … often feel responsible for that and guilty.

(Wanda, Worker 11)

Another participant suggested that the concepts of outreach and stigma were to some extent influenced by wider societal perceptions about what constitutes a carer and a person in need of ‘care’:

I think the difficulty that we have is … that [eating disorders are] currently seen as … only affect[ing] middle class or well-to-do girls.

(Marcus, Vol 4)

The concepts of stigma and mistrust are inter-related and carers' workers were particularly concerned that better identification could only take place in a context of trust. ‘Hidden’ carers would not come forward unless they thought that they would be treated fairly and in accordance with their wishes. For example, experiences of discrimination in the past meant that gay, lesbian, bisexual or transgender carers would not disclose their sexual orientation or sexual identity immediately (Guasp 2011):

People don't come in the first instance and say, ‘I'm gay’ or ‘I'm transgender’. They come in and they talk about their partner who is ill and then they say, ‘Can you come and visit?’

(Kathleen, Vol 13)

### Outreach with other professionals

The final theme relates to the extent to which outreach is not simply about carers finding out information and asking for support, but is also dependent upon practitioners' ability to identify carers and explain to them what help is available:

Case by case, individual social workers are pretty good at identifying [carers] when they do come into contact with the family, so I know by default we've almost [always] got contact with those people.

(Delia, Commissioner 6)

In a different study area, a worker with a specific role to support carers was less convinced of practitioners' ability to identity carers in need of support:

There's the outreach work going out to speak to different [social work] teams. With some teams, it is like bashing my head against a brick wall … Very difficult to do. The culture is sometimes really hard set, so it's a case of going out there and keep … beating the drum.

(Candy, Worker 15)

## Discussion

It is important to preface this discussion by acknowledging the study limitations. This was exploratory research with a broad remit that extended beyond outreach. As such, it can only make suggestions for possible avenues for further work and cannot comment on the effectiveness of any of the models described above. Consistent with the large body of research on carers (e.g. Katbamna *et al.* 2004, Parker *et al.* 2010, Carers UK 2013), a number of barriers exist that prevent carers from seeking support. This study found, as have many others (e.g. O'Connor 2007), that these barriers often exist because many carers do not recognise themselves as a carer and because feelings such as guilt and stigma may inhibit people from asking for support. Along with Copello and Templeton (2012), this is one of the few studies to highlight the way in which carers of people who misuse substances may experience these feelings.

However, the study also shows that the process is not simply one of carers ‘recognising’ that they are carers; it is also about how carers are identified and recognised by others. For instance, we know very little about what social workers and other social and healthcare practitioners do in these circumstances. It is striking that criticisms of the social work qualifying curriculum from employers, politicians and policy makers focus so much on what newly qualified social workers know about child development or communication with children (Moriarty & Manthorpe 2014), yet so little is known about what they, and other social and healthcare practitioners are taught about identifying and supporting carers.

Changes to information technology have meant that it is increasingly expected that people will access social and healthcare information online. Debates on this topic are usually framed in terms of the ‘digital divide’ and differences between those who can use and have access to the internet, and those who do not. However, it is rare to question the relevance and quality of the information that is provided this way, even though it appears to be very variable (Manthorpe *et al.* 2013). There is also a need to think more about the circumstances in which carers access such information. At times of crisis or when carers have had to increase the amount of care they provide very suddenly, they are unlikely to have the time to look at it in detail.

An important question raised by this study is how councils should seek to balance their resources between generic information aimed at all carers in their locality, regardless of the intensity of the support that they provide and their responses to it, and the resources allocated to help carers who qualify for social care support. If too much emphasis is placed on the former, then resources may be spread too thinly. Without greater attention to how information is used and more consideration of its relevance, there is a risk that some carers will remain excluded. As the changes created by the Care Act 2014 are implemented, local councils will need to consider if better outreach for carers might assist them in meeting the government's aim of preventing or delaying carers' needs for support.

## Abbreviations

INT: If you wanted more information, would you look on the local authority website?
RES: I wouldn't now, because [daughter's] all right. It's when she's not all right that I need somebody.
INT: Where would you look if you wanted information?
RES: I don't know. I just wouldn't think of it. I think I'm in too much of a state [then] to even use the computer to be honest. (Wilma, Carer 22)
